# Noise insensitive volumetric fusion method for enhanced photoacoustic microscopy

**DOI:** 10.1117/1.JBO.28.10.106501

**Published:** 2023-10-04

**Authors:** Sihang Li, Hao Wu, Hongyu Zhang, Zhipeng Zhang, Qiugen Liu, Xianlin Song

**Affiliations:** a Nanchang University, School of Information Engineering, Nanchang, China; b Nanchang University, Jiluan Academy, Nanchang, China

**Keywords:** photoacoustic microscopy, noise insensitive, volumetric fusion, spatial domain, extended depth-of-field

## Abstract

**Significance:**

Photoacoustic imaging is an emerging imaging modality that combines the high contrast of optical imaging and the high penetration of acoustic imaging. However, the strong focusing of the laser beam in optical-resolution photoacoustic microscopy (OR-PAM) leads to a limited depth-of-field (DoF).

**Aim:**

Here, a volumetric photoacoustic information fusion method was proposed to achieve large volumetric photoacoustic imaging at low cost.

**Approach:**

First, the initial decision map was built through the focus detection based on the proposed three-dimensional Laplacian operator. Majority filter-based consistency verification and Gaussian filter-based map smoothing were then utilized to generate the final decision map for the construction of photoacoustic imaging with extended DoF.

**Results:**

The performance of the proposed method was tested to show that our method can expand the limited DoF by a factor of 1.7 without the sacrifice of lateral resolution. Four sets of multi-focus vessel data at different noise levels were fused to verify the effectiveness and robustness of the proposed method.

**Conclusions:**

The proposed method can efficiently extend the DoF of OR-PAM under different noise levels.

## 1 Introduction

Photoacoustic imaging, which combines the advantages of optical imaging and acoustic imaging to provide high-resolution and non-invasive imaging with deep penetration depth,^1^^–^^6^ has been widely applied in biomedicine, such as breast cancer diagnosis,^7^ thyroid imaging,^8^ and brain imaging.^9^ As an important branch of photoacoustic imaging, optical-resolution photoacoustic microscopy (OR-PAM) satisfies the criterion of high-resolution imaging in biomedical research.^10^ Raster scanning is utilized in OR-PAM to capture three-dimensional (3D) information. However, the reliance on a focused laser beam for high-resolution imaging introduces challenges, such as reduced lateral resolution outside the focal regions and a limited depth-of-field (DoF). The restricted DoF in OR-PAM consequently hampers volumetric imaging speed, thereby imposing limitations on its practical applications, such as imaging of biological tissue with a rough surface (e.g., cerebrovascular^11^) and fast acquisition of physiological and pathological processes.^7^^,^^9^ Conventional photoacoustic microscopy utilizes axial scanning to achieve the volumetric imaging of sample, and the multi-focus photoacoustic data can be acquired by mechanically moving the probe or sample.^12^ The utilization of volumetric fusion of multi-focus photoacoustic data is a cost-effective strategy for enhancing the DoF of OR-PAM.

To address the limited DoF of OR-PAM, Yao et al.^13^ proposed double-illumination photoacoustic microscopy (PAM) by illuminating the sample from both the top and bottom sides simultaneously, which provides improved penetration depth and extended DoF. However, this method is restricted to thin biological tissue. Shi et al.^14^ utilized the Grueneisen relaxation effect to suppress the artifact introduced by the sidelobe of Bessel beam to achieve PAM with extended DoF. However, two lasers are required to excite the nonlinear photoacoustic signal. Hajireza et al.^15^ reported a multifocus OR-PAM for extended DoF based on wavelength tuning and chromatic aberration. However, this system is limited to the acquisition of multifocus imaging at discrete depths. These methods can achieve high-resolution photoacoustic imaging with large DoF, at the expense of increased system complexity and high cost. Multi-focus image fusion (MFIF), which is used to integrate multiple images of the same target with different focal positions into single in-focus image,^16^^–^^18^ has shown promising prospects in addressing the narrow DoF of microscopy system recently.^19^^,^^20^ Awasthi et al.^21^ proposed a deep learning-based model for fusing the photoacoustic images reconstructed using different algorithms to improve the quality of photoacoustic imaging. However, this model is primarily targeted at photoacoustic tomography and a large amount of data is required for training. Zhou et al.^22^ utilized a 2D image fusion algorithm with enhancement filtering to construct the photoacoustic image with extended DoF for accurate vascular quantification. However, this method falls short in the fusion of volumetric information for photoacoustic data.

In this work, a cost-effective volumetric fusion method is proposed, to facilitate the acquisition of high-resolution and large volumetric photoacoustic image with conventional PAM. The focused regions in multi-focus photoacoustic data were identified with the proposed 3D modified Laplacian operator. The misidentified regions in the built initial decision map (IDM) were corrected by consistency verification, and Gaussian filter (GF) was employed to smooth the map and reduce block artifact. Finally, photoacoustic data with enhanced DoF can be achieved by the voxel-wise weighted-averaging based on the final decision map (FDM). Quantitative evaluation suggests that the DoF of photoacoustic microscopy can be expanded by a factor of 1.7 while maintaining the lateral resolution within focused regions through the proposed method. The effectiveness and robustness of the proposed method were verified by fusing four sets of multi-focus vessel data under different noise levels.

## 2 Method

### 2.1 Volumetric Fusion Based on 3D Modified Laplacian Operator

To construct high-resolution and large volumetric photoacoustic imaging, the focused regions in multi-focus photoacoustic data were extracted and preserved in the fused imaging. A focus measure based on 3D modified Laplacian operator, which quantifies the sharpness of photoacoustic imaging, was proposed to identify focused regions within multi-focus data. The Laplacian operator $\nabla^{2}$ for photoacoustic data P is defined as

$$\nabla^{2}P=\frac{\partial^{2}P}{\partial x^{2}}+\frac{\partial^{2}P}{\partial y^{2}}+\frac{\partial^{2}P}{\partial z^{2}}.$$ (1)

The second derivatives in orthogonal directions can have opposite signs and cancel each other.^23^ The 3D modified Laplacian operator $\nabla_{M}^{2}$ for photoacoustic data P, which utilizes the absolute values of the second derivatives to measure the signal intensity variation, is defined as

$$\nabla_{M}^{2}P=|\frac{\partial^{2}P}{\partial x^{2}}|+|\frac{\partial^{2}P}{\partial y^{2}}|+|\frac{\partial^{2}P}{\partial z^{2}}|,$$ (2)

The sharper edges and contours within DoF, which result in more rapid intensity variation of photoacoustic signal, give higher response to the modified Laplacian operator. ML is defined as the discrete approximation of the modified Laplacian operator $\nabla_{M}^{2}$. The ML for photoacoustic data P is given by

$${\mathrm{ML}}_{P}(x,y,z)=|2P(x,y,z)-P(x-1,y,z)-P(x+1,y,z)|\;+|2P(x,y,z)-P(x,y-1,z)-P(x,y+1,z)|\;+|2P(x,y,z)-P(x,y,z-1)-P(x,y,z+1)|,$$ (3)

where P(x,y,z) is the signal intensity of P at (x,y,z). The focus measure for the i’th image block of P centered at $\left(x_{0},y_{0},z_{0}\right)$ is defined as the sum-modified Laplacian (SML) within the i’th block as shown in Eq. (4):

$${\mathrm{SML}}_{P}^{i}=\overset{x=x_{0}+N}{\underset{x=x_{0}-N}{\sum }}\overset{y=y_{0}+N}{\underset{y=y_{0}-N}{\sum }}\overset{z=z_{0}+N}{\underset{z=z_{0}-N}{\sum }}{\mathrm{ML}}_{P}(x,y,z),$$ (4)

where N determines the size of the block. The SML evaluates the high frequency information of an image block, and a larger SML represents a higher level of focus. The multi-focus photoacoustic data $P_{1}$ and $P_{2}$ with the size of H×W×L simulated through the virtual OR-PAM^24^ were divided into non-overlapping blocks with equal size of $(2N+1{)}^{3}$, respectively. The focus measures based on the 3D modified Laplacian operator for each block in $P_{1}$ and $P_{2}$ were computed to build the IDM as shown in Eq. (5):

$$\mathrm{IDM}(x,y,z)=\{\begin{array}{ll} 1 & {\mathrm{SML}}_{P_{1}}^{i}>{\mathrm{SML}}_{P_{2}}^{i}\\ 0 & {\mathrm{SML}}_{P_{1}}^{i}{<\mathrm{SML}}_{P_{2}}^{i}\\ 0.5 & {\mathrm{SML}}_{P_{1}}^{i}={\mathrm{SML}}_{P_{2}}^{i} \end{array},$$ (5)

where ${\mathrm{SML}}_{P_{1}}^{i}$ and ${\mathrm{SML}}_{P_{2}}^{i}$ are the focus measures for the i’th block in $P_{1}$ and $P_{2}$, respectively. The voxels within focused regions in multi-focus photoacoustic data can be identified through IDM. The voxel (x,y,z) is considered to be within the focused regions in $P_{1}$ if IDM (x,y,z)=1, and be within the focused regions in $P_{2}$ if IDM (x,y,z)=0. The noise in the photoacoustic data can cause errors in the process of focus detection. Therefore, the consistency verification based on majority filter (MF) was employed to refine the IDM. If the j’th block is identified as the focused region in $P_{1}$ while the adjacent six blocks in the orthonormal six directions are identified as the focused regions in $P_{2}$, the IDM for the voxels of *j*’th block are switched to zero and vice versa. The GF was then employed on the refined IDM to smooth the boundaries to generate FDM. The Gaussian filtering for IDM is formulated as

$$\mathrm{FDM}(x,y,z)=\frac{1}{W}\underset{(x^{\prime },y\prime ,z\prime )\in S}{\sum }G(x^{\prime },y\prime ,z\prime ,x,y,z)\mathrm{IDM}(x^{\prime },y\prime ,z\prime ),$$ (6)

where G is the Gaussian function for spatial difference. S is a window centered at (x,y,z). W is the normalization factor defined as

$$W=\underset{(x^{\prime },y\prime ,z\prime )\in S}{\sum }G(x^{\prime },y\prime ,z\prime ,x,y,z),$$ (7)

where G is given by Eq. (8),

$$G(x^{\prime },y\prime ,z\prime ,x,y,z)=\exp(-\frac{(x-x^{\prime }{)}^{2}+(y-y\prime {)}^{2}+(z-z\prime {)}^{2}}{2\sigma^{2}}),$$ (8)

where σ is the standard deviation of Gaussian function G. The high-resolution and large volumetric photoacoustic imaging $P_{f}$ was computed by the voxel-wise weighted-averaging as shown in Eq. (9):

$$P_{f}(x,y,z)=\mathrm{FDM}(x,y,z)P_{1}(x,y,z)+(1-\mathrm{FDM}(x,y,z))P_{2}(x,y,z).$$ (9)

**Fig. 1:**
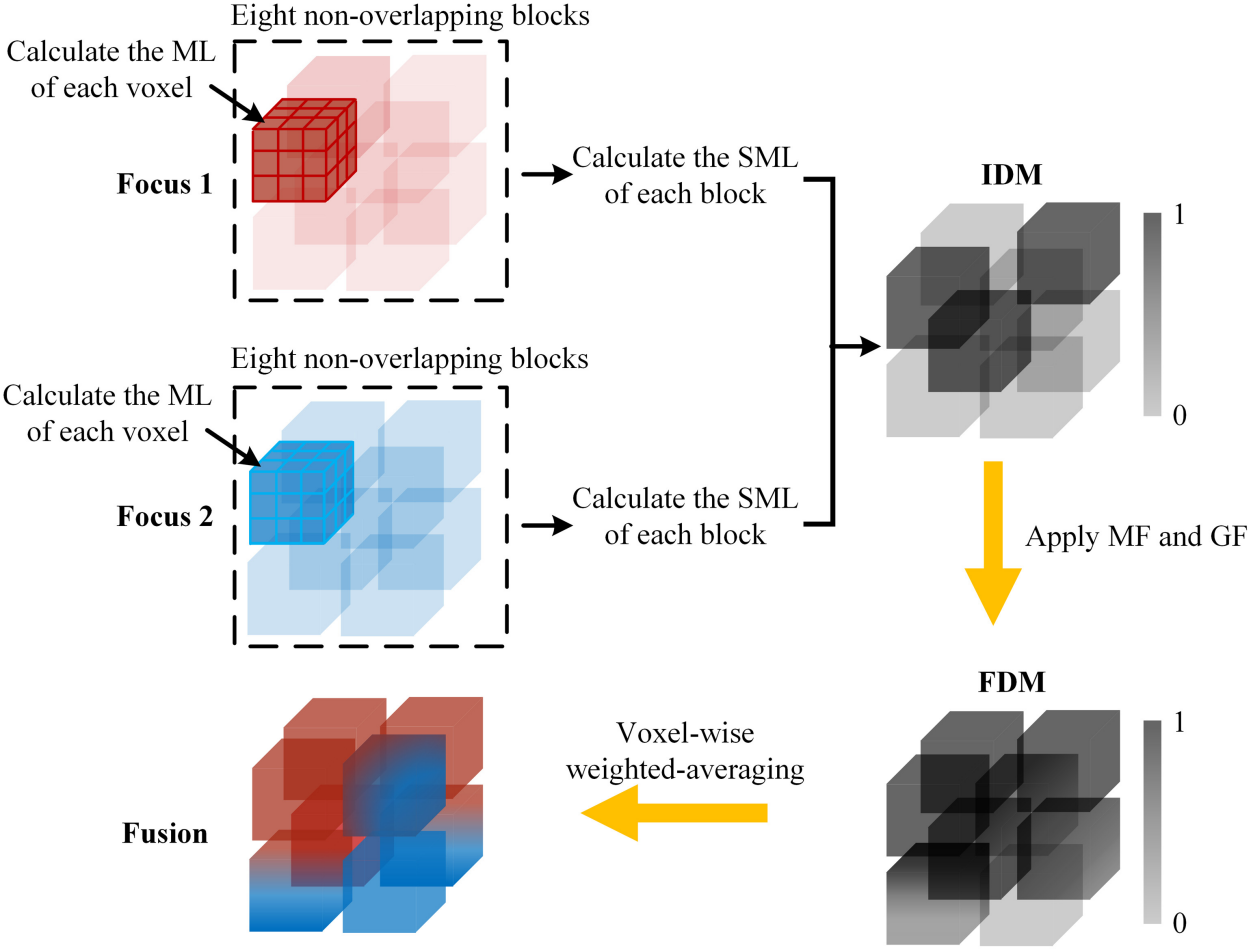
Proposed volumetric fusion method based on SML. ML is the discrete approximation of $\nabla_{M}^{2}$.

The process of the proposed volumetric fusion method is shown in Fig. 1. The ML of each voxel in multi-focus photoacoustic imaging was computed, and the multi-focus photoacoustic imaging was divided into non-overlapping blocks. The SML of each block was calculated and compared to construct the IDM. The IDM is refined with MF and smoothed with GF to generate the FDM. The Fusion can be constructed by voxel-wise weighted-averaging of multi-focus imaging based on the FDM.

### 2.2 Multi-Focus 3D Data Simulation through Virtual OR-PAM

**Fig. 2:**
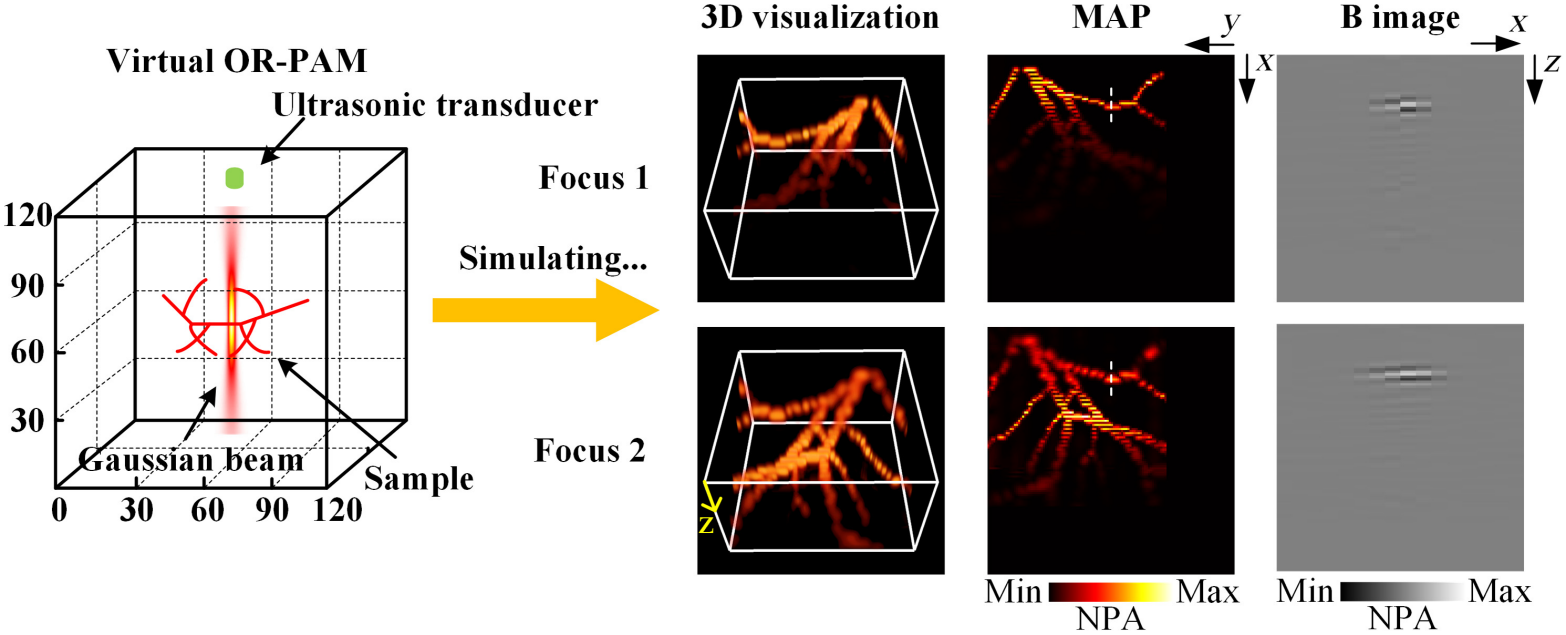
Acquisition of multi-focus photoacoustic imaging through the virtual OR-PAM. NPA, normalized photoacoustic amplitude.

The multi-focus 3D photoacoustic data were simulated through a virtual photoacoustic microscopy^24^ using Gaussian beam, as shown in Fig. 2. An objective lens with a numerical aperture of 0.14 was used to generate the Gaussian beam. The wavelength of light was set as 532 nm. The 3D grid is Nx×Ny×Nz=120×120×120 and the pixel size is dx=dy=2  μm, dz=3  μm. The medium around the sample was set as water, and the speed of sound was set to 1500 m/s. The photoacoustic signal was collected using an ultrasonic detector with a center frequency of 75 MHz and a bandwidth of 67%. Multi-focus photoacoustic data with two focuses were employed as an example to demonstrate the proposed method. Two vertically tilted fibers were placed in the grid as required and imaged to test the performance of the proposed method. Four sets of multi-focus tilted vessel at five noise levels (Gaussian noise was added in the experiment since most noise in photoacoustic imaging can be considered as Gaussian noise^25^^–^^28^) were simulated to further validate the robustness and effectiveness of the proposed method. The experiment data in this work were simulated through a 64-bit Windows 10, Intel (R) Core (TM) i7-12700H CPU @ 2.30 GHz desktop running windows operating system. The 3D visualization and max amplitude projection (MAP) images of the simulated multi-focus photoacoustic data presented in Fig. 2 show that the imaging within DoF reveals more details while the imaging outside the DoF appears partially blurred. The B images of the simulated multi-focus data at the position indicated by the white dashed lines in the MAP images demonstrate that the lateral resolution within focused regions is better than that of the defocused regions.

## 3 Results

### 3.1 Performance Test by Fusing Multi-Focus Vertically Tilted Fiber

**Fig. 3:**
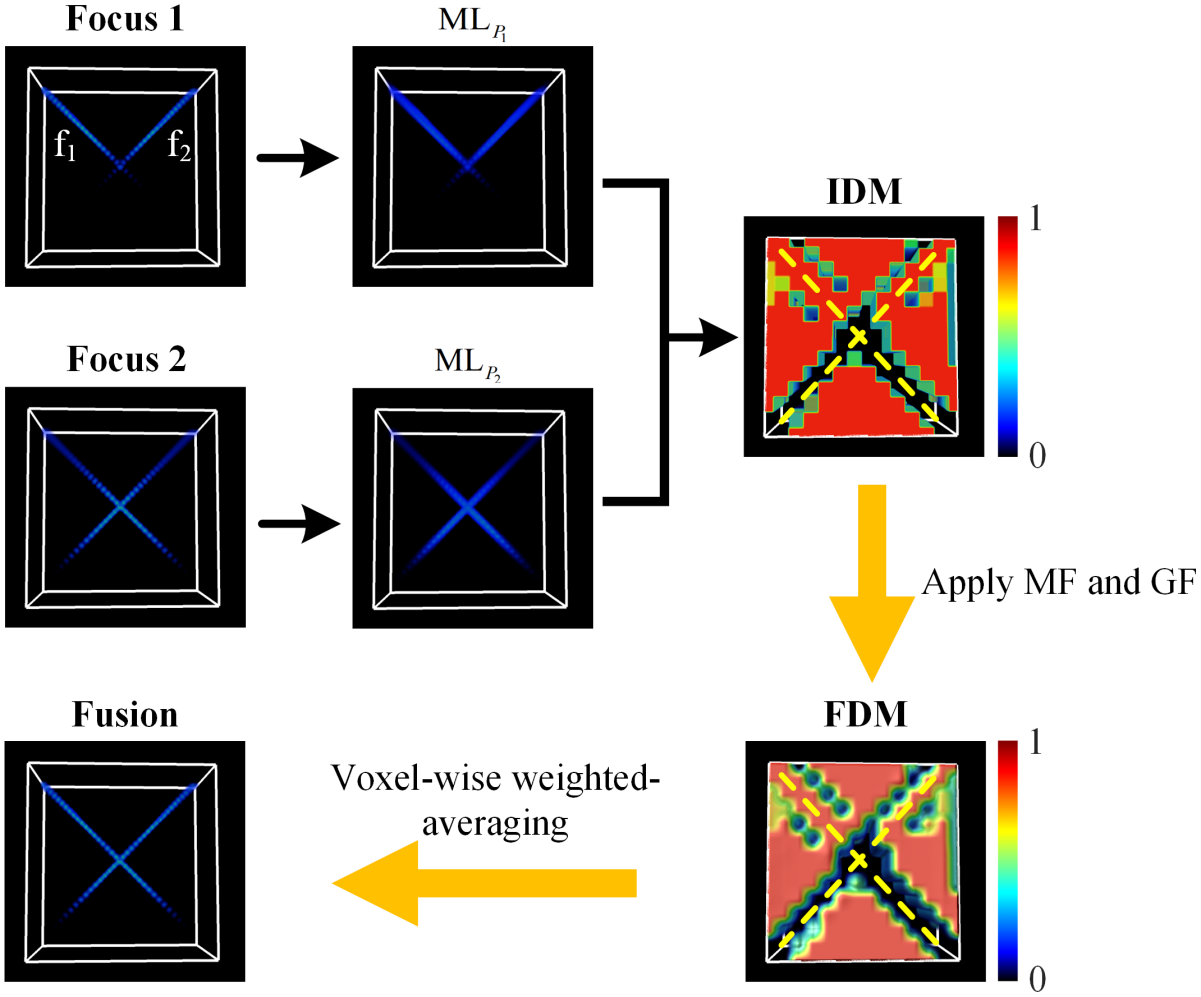
Demonstration for the process of volumetric fusion, taking the fusion of multi-focus fiber as an example. $f_{1}$ and $f_{2}$ are the two vertically tilted fibers. ${\mathrm{ML}}_{P_{1}}$ and ${\mathrm{ML}}_{P_{2}}$ are the discrete approximation of $\nabla_{M}^{2}$ for $P_{1}$ and$P_{2}$, respectively. The yellow dashed lines in IDM and FDM indicate the position of fibers $f_{1}$ and $f_{2}$.

**Fig. 4:**
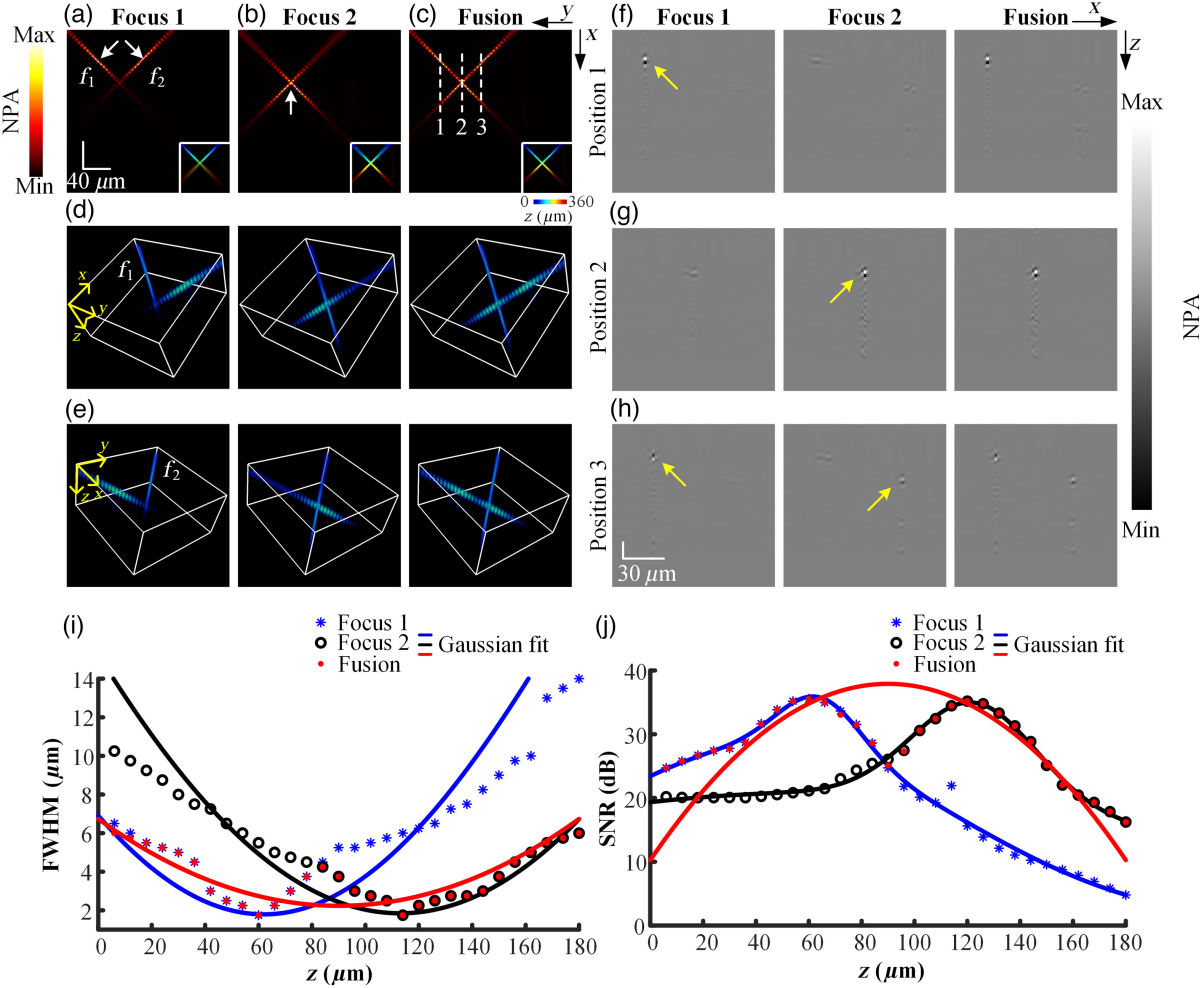
(a), (b) MAP images of multi-focus fiber data. (c) MAP image of the fiber fused through the proposed method. The depth-coding MAP images of (a)–(c) are presented in the lower right corner, respectively. (d), (e) 3D visualization of the multi-focus fiber from two views, respectively. $f_{1}$ and $f_{2}$ are the two vertically tilted fibers. (f)–(h) B images of the white dashed lines in panel (c) before and after fusion. (i) Variation of FWHM along with the depth before and after fusion. (j) Variation of SNR along with the depth before and after fusion. NPA, normalized photoacoustic amplitude.

The performance of the proposed method was tested by fusing multi-focus vertically tilted fibers as shown in Figs. 3 and 4. Figure 3 shows the process of the proposed volumetric information fusion method, taking the simulated fibers $f_{1}$ and $f_{2}$ as an example. The focus measures based on 3D modified Laplacian operator of multi-focus fiber were calculated to generate the IDM. The IDM was then refined and smoothed by filtering to generate the FDM, and photoacoustic imaging with extended DoF can be achieved by the voxel-wise weighted-averaging, as shown in Fig. 3.

Figures 4(a) and 4(b) are the MAP images of multi-focus fiber where the optical focuses were set at z=40 (Focus 1) and z=60 (Focus 2) in the 3D grid, respectively. Figure 4(c) is the MAP image of the fused fiber (Fusion). The depth-coding MAP images of Figs. 4(a)–4(c) are displayed in the lower right corner, respectively. The focal planes of Focus 1 and Focus 2 are indicated by the white arrows. Figures 4(d) and 4(e) are the 3D visualization of the fibers before and after fusion from two views rendered by Amira software, respectively. The narrow DoF limits OR-PAM from capturing the complete structure of fibers through single imaging. The lateral resolution and signal-to-noise ratio (SNR) degrade rapidly outside the focal plane, which results in partially blurred imaging. The location of optical focus determines the clear portion of imaging. As shown in Figs. 4(a)–4(c), the large volumetric and high-resolution fiber can be achieved by fusing multi-focus fiber data through the proposed method. The B images of Focus 1, Focus 2, and Fusion at three depths indicated by the white dashed lines in Fig. 4(c) are shown in Figs. 4(f)–4(h). The in-focus signals in the B images are indicated by the yellow arrows. The lateral resolution of the focused regions can be preserved in the fused B images, which verifies that the proposed method can identify the focused regions accurately at different depths. The full width at half maximum (FWHM) of the profile of the fiber $f_{1}$ before and after fusion was measured, as shown in Fig. 4(i). A smaller FWHM suggests a better lateral resolution. The lateral resolution in the focused part (30 to 100  μm of Focus 1, 80 to 150  μm of Focus 2) is better than that of the defocused part (80 to 150  μm of Focus 1, 30 to 100  μm of Focus 2). The DoF of OR-PAM is quantified as the depth interval over which the FWHM of the fiber becomes twice that of the focal plane. The DoF of the fiber of Focus 1, Focus 2, and Fusion was measured to be about 71.2, 79.9, and 124.6  μm, respectively, which suggests that the proposed method can increase the DoF of OR-PAM by a factor of 1.7 without sacrificing the lateral resolution. The SNR variation of the fiber $f_{1}$ along the depth direction was measured, as shown in Fig. 4(j). The SNR in the focused part (30 to 100  μm of Focus 1, 80 to 150  μm of Focus 2) is higher than that of the defocused part (80 to 150  μm of Focus 1, 30 to 100  μm of Focus 2) and is precisely preserved in the fused fiber.

### 3.2 Large Imaging of Vascular

**Fig. 5:**
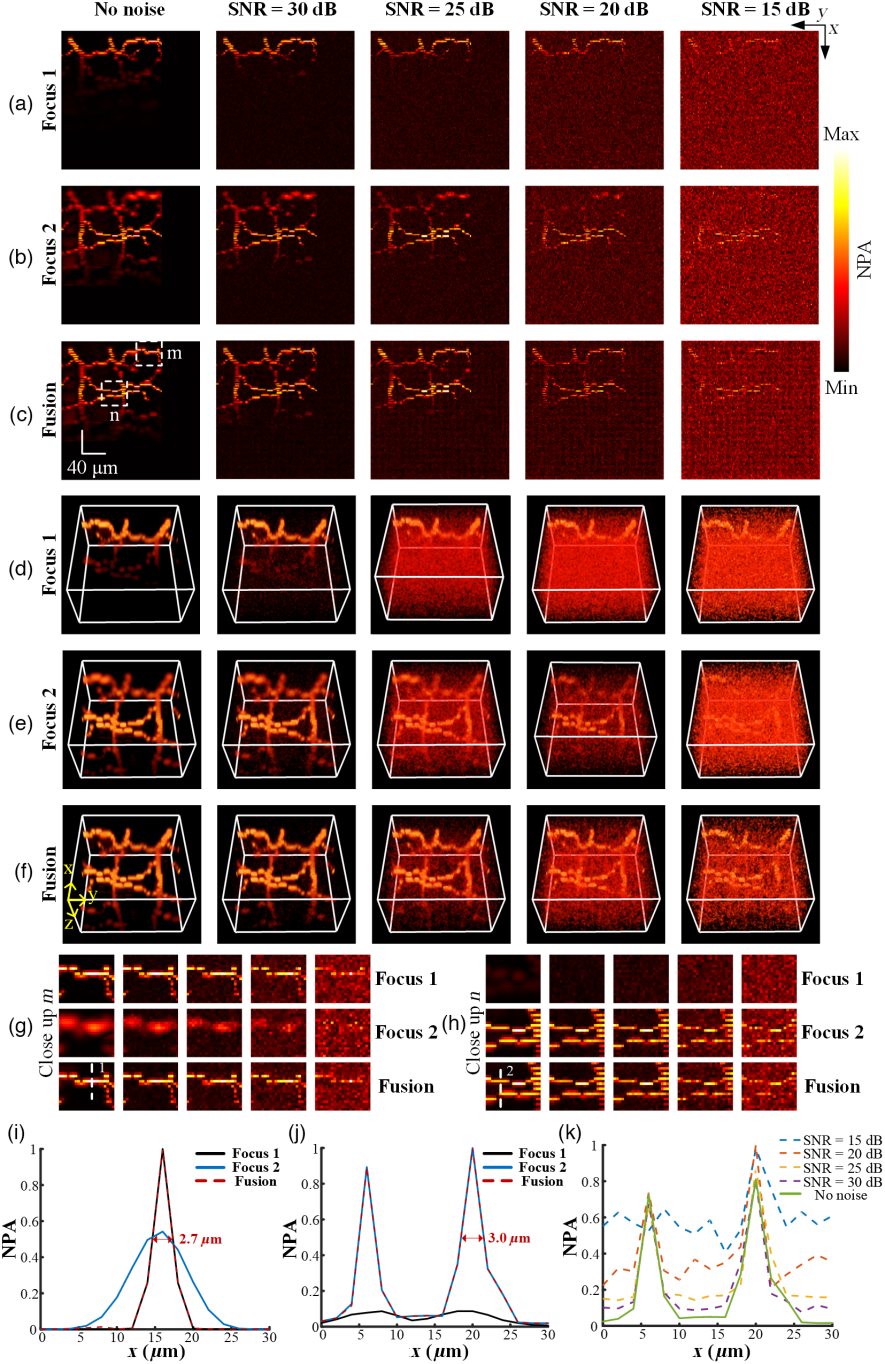
(a)–(b) MAP images of multi-focus vessel data at five noise levels. (c) MAP image of the vessel fused through the proposed method. (d)–(f) 3D visualization for (a)–(c) rendered by the Amira software. (g) Close-up images of vessel before and after fusion at five noise levels indicated by the white dashed rectangle m in panel (c). (h) Close-up images of vessel before and after fusion at five noise levels indicated by the white dashed rectangle n in panel (c). (i), (j) Normalized intensity distribution before and after fusion at position 1 and 2 indicated by the white dashed lines in panels (g) and (h). (k) Normalized intensity distribution of the fused vessel under five noise levels at position 2 indicated by the white dashed line in panel (h). NPA, normalized photoacoustic amplitude.

The robustness and effectiveness of the proposed method were verified by fusing multi-focus vessels at five noise levels, as shown in Fig. 5. Figures 5(a) and 5(b) are the MAP images of 1 set of multi-focus vessels where the optical focuses were set at z=35 (Focus 1) and z=60 (Focus 2) in the 3D grid, respectively. The complete structure of the vessel cannot be captured in single imaging due to the narrow DoF as shown in Figs. 4(a) and 4(b). The noise presented in the MAP images increases with the decrease in SNR. Figure 5(c) is the MAP image of the high-resolution and large volumetric data (Fusion) obtained via the proposed method at five noise levels, which verifies the remarkable robustness to noise using our method. The focused regions can be accurately identified through the proposed 3D modified Laplacian operator under noise condition. Figures 5(d)–5(f) are the 3D visualization rendered by Amira software for intuitive observation. The normalized intensity distribution at positions 1 and 2 indicated by the white dashed lines in Figs. 5(g) and 5(h) was analyzed to evaluate the capability to preserve lateral resolution within focused regions using our method, as shown in Figs. 5(i) and 5(j). When there is no noise, the FWHM of the normalized photoacoustic signals of Focus 1, Focus 2, and Fusion at position 1 was measured to be 2.7 (Focus 1), 9.3 (Focus 2), and 2.7  μm (Fusion), respectively, as shown in Fig. 5(i). The FWHM of the second peak of the normalized photoacoustic signals of Focus 1, Focus 2, and Fusion at position 2 was measured to be 9.9 (Focus 1), 3.0 (Focus 2), and 3.0  μm (Fusion), respectively, as shown in Fig. 5(j). The lateral resolution within focused regions can be maintained in the fused vessel through the voxel-wise weighted-averaging fusion rule, which validates the effectiveness of the proposed method in processing the sample with intricate structure. The normalized intensity distribution of the Fusion at position 2 under different noise levels was analyzed, as shown in Fig. 5(k). The influence of noise on the photoacoustic signal is insignificant when SNR is 30 dB. The decrease in SNR leads to the increase of the influence of noise on the photoacoustic signal. The photoacoustic signal cannot be visually distinguished from the added noise when SNR drops to 15 dB, as shown in Fig. 5(k). However, the focused regions within DoF can be accurately identified and preserved through the proposed method when a high level of noise is added, which further verifies the effectiveness and robustness of our method.

**Fig. 6:**
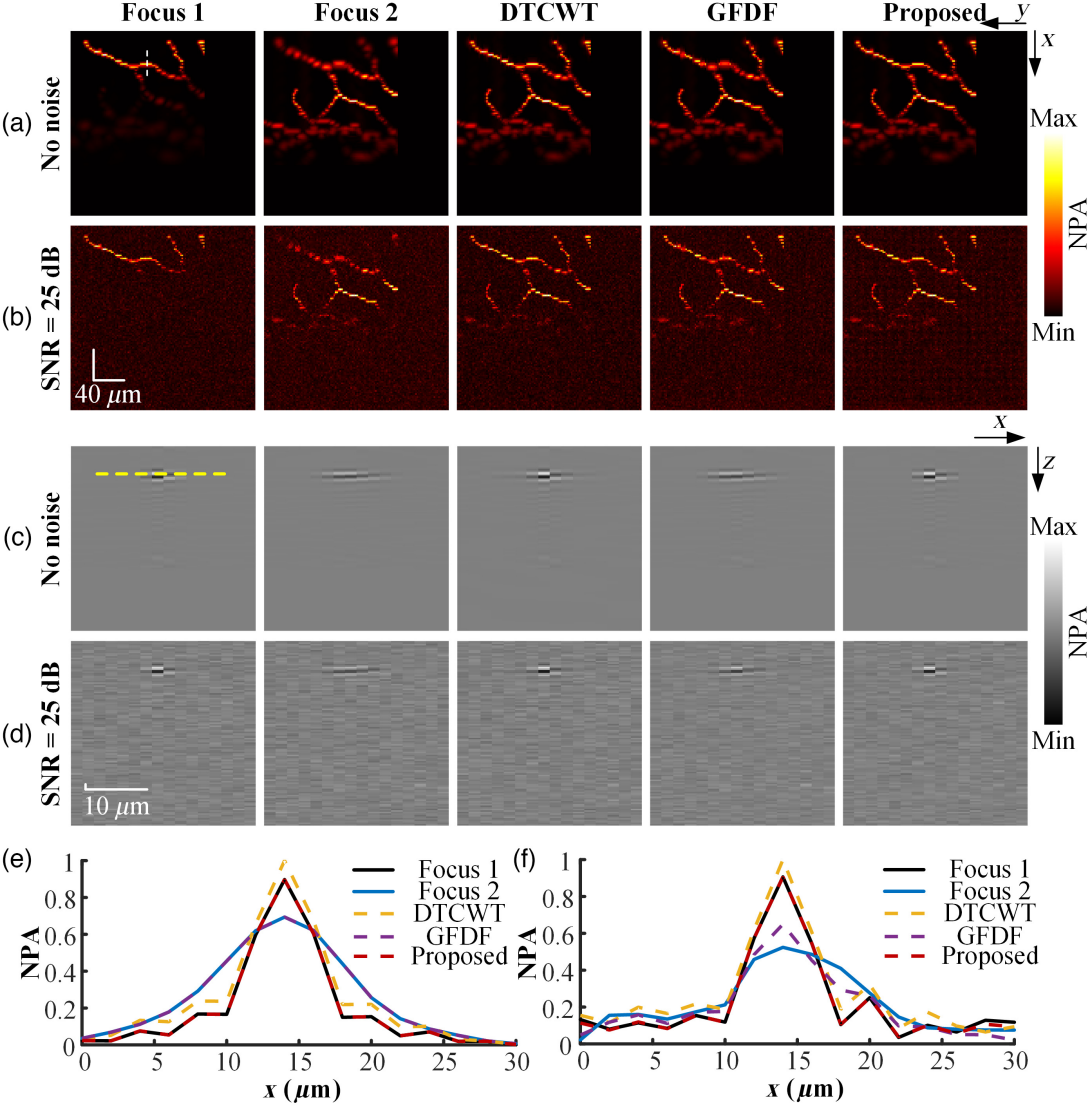
(a), (b) MAP images of Focus 1, Focus 2, and the fused data when there is no noise and SNR=25  dB, respectively. (c), (d) B images at the position indicated by the white dashed line in panel (a) when there is no noise and SNR=25  dB, respectively. (e), (f) Normalized intensity distribution at the position indicated by the yellow dash line in panel (c) when there is no noise and SNR=25  dB, respectively. NPA, normalized photoacoustic amplitude.

The superior performance of the proposed method over previous representative 2D-based MFIF algorithms was verified by comparing the MAP images and B images of the fused data. Two state-of-the-art MFIF methods, including the transform domain-based method dual tree complex wavelet transform (DTCWT)^29^ and the spatial domain-based method guided filter-based focus region detection for multi-focus image fusion (GFDF),^30^ were selected for comparison. Four common metrics in MFIF were selected to quantify the performance of different methods from multiple perspectives, including (1) information theory-based metric cross entropy (CE),^31^ which estimates the dissimilarity between source images and fused image in terms of information; (2) image feature-based metric spatial frequency (SF),^32^ which reveals the edge and texture information of the fused image; (3) human perception-based metric $Q_{\mathrm{CV}}$,^33^ which quantifies the performance of MFIF algorithm by leveraging the principles of human visual system; and (4) similarity-based metric structural similarity index measure (SSIM),^34^ which measures the similarity between source images and fused image in terms of luminance, contrast, and structure. The multi-focus volumetric imaging of vessel was sliced to establish the multi-focus slice sequence. The 2D slices at the same position in multi-focus sequence are processed with DTCWT and GFDF, respectively. The fused 2D slices were stacked to produce high-resolution photoacoustic imaging with extended DoF. As shown in Fig. 6, one group of simulated multi-focus vessel was selected to compare different methods at two noise levels. Figures 6(a) and 6(b) are the MAP images of the Focus 1, Focus 2, and fused vessel obtained via different methods when no noise is added and SNR=25  dB, respectively. The B images at the position indicated by the white dashed line in Fig. 6(a) before and after fusion were compared, as shown in Figs. 6(c) and 6(d). The normalized intensity distribution of photoacoustic signal processed with Hilbert transform at the position indicated by the yellow dashed line in Fig. 6(c) before and after fusion is compared, as shown in Figs. 6(e) and 6(f). The proposed method, which utilizes the 3D modified Laplacian operator for the focus measure of volumetric imaging, can accurately identify and preserve the lateral resolution within focused regions at different noise levels compared to 2D-based MFIF methods. By contrast, the GFDF, which was affected by the lateral resolution outside the DoF in Focus 2, failed to identify the lateral resolution within focused regions at different noise levels. The poorer lateral resolution within the defocused regions in Focus 2 was mistakenly preserved in the fused photoacoustic imaging, as shown in Figs. 6(c)–6(f). The outperformance of the proposed method is attributed to the direct focus detection and fusion of volumetric information, whereas the slicing process of volumetric imaging leads to a loss of spatial correlation when implementing 2D-based MFIF methods. The MAP images of the 4 groups of high-resolution and large volumetric vessel obtained through different methods were evaluated using 4 metrics when there is no noise and SNR=25  dB, respectively, as shown in Table 1. The proposed volumetric fusion method outperforms the conventional 2D-based MFIF method from multiple perspectives, which further validates the effectiveness of the direct fusion of volumetric photoacoustic information.

**Table 1:** Quantitative evaluation of different methods.

Method	Noise level	CE	SF	$Q_{\mathrm{CV}}$	SSIM
DTCWT	No noise	0.1008	28.24	34.42	1.7263
	25 dB	0.1200	33.92	44.17	1.4280
GFDF	No noise	0.0977	27.11	140.85	1.7241
	25 dB	0.1012	33.10	132.11	1.4412
Proposed	No noise	0.0863	28.82	25.37	1.7264
	25 dB	0.0976	34.39	35.96	1.4277

## 4 Conclusion and Discussion

We proposed a noise insensitive volumetric fusion method that utilizes 3D modified Laplacian operator and Gaussian filtering to enhance the DoF of OR-PAM. Experimental results demonstrate that the proposed method is capable of extending the DoF of OR-PAM by a factor of 1.7 and shows superior performance at different levels of noise. The superiority of the proposed method over previous 2D-based MFIF methods was quantitatively verified with four categories of metrics. Our work provides a cost-effective approach for the acquisition of photoacoustic imaging with extended DoF.

The virtual OR-PAM, which is capable of performing A, B, and C scan, was verified to be consistent with the actual OR-PAM system.^11^^,^^35^^,^^36^ Hence, the experiments based on the virtual OR-PAM are reliable. For sample with simple structure, the focused boundary can be determined through the quantification of FWHM or SNR, and the volumetric fusion can be achieved through the simple combination of multi-focus data. For example, the focused boundary of the multi-focus fiber in this work can be estimated as a single plane given by the intersection between the FWHM of Focus 1 and Focus 2, as shown in Fig. 4(i). For sample with intricate structure (such as cerebrovascular), accurately quantifying the variation of FWHM and SNR along depth direction is difficult. In addition, the depth of optical focus experiences a shift due to the variations in scattering and absorption of heterogeneous samples.^37^ The focused boundary cannot be approximated as a single plane. Therefore, this approach is not applicable to turbid biological tissue and limited to transparent sample with weak absorption and scattering such as water. Furthermore, this approach can be time-consuming and labor-intensive for multi-focus data that include more than two focuses. By contrast, the proposed method can automatically identify and preserve the focused regions within multi-focus data in the fusion results.

In this work, the effectiveness of the proposed method was demonstrated through dual-focus photoacoustic data of fiber and vessel. Actually, the proposed method can be applied to multi-focus data that include more than two focuses by pairwise fusion. Dual-focus data with adjacent focuses can be first combined through the proposed method. Then, the resulting fused data can be subsequently integrated with data from another adjacent focus. This process is repeated iteratively until the data from all focuses have been processed to achieve high-resolution and large volumetric photoacoustic imaging. The proposed method is not limited by the focal positions, or the number of focuses in multi-focus data. Compared to the approach of estimating a focused boundary through FWHM quantification, the proposed method exhibits the advantages of enhanced flexibility, ease of portability, and broader applicability.

## Data Availability

The data that support the findings of this study are available upon reasonable request.
